# An Influenza Outbreak

**Published:** 1918-11

**Authors:** 


**An Influenza Outbreak.** By C. W. WIRGMAN, M. D. Lond., F. R. C. S. Eng. *The Lancet*, Sept. 7, 1918.

Among 516 men in one camp, 149 were reported ill with influenza between June 25 and July 6. At first the younger men fell ill, while as time went on the older ones were struck down by the disease.

The cases did not prove severe; only one slight attack of bronchitis occurred.

The general symptoms were headache, pains in back and limbs, oreness in eyes, sore-throat and cough. The tongue was generally heavily coated. Temperature rose to 104° F., but, as a rule, after three days it was below 99°. The average stay in the hospital was 3 1/2 days.

Rest in bed proved a very efficient treatment.

As to methods of prophylaxis, nasal lavages and gargles with normal saline were carried out twice a day, and confined atmospheres avoided as much as possible.

